# Editorial: Metabolic response: nexus or nemesis for the understanding of sports nutrition and doping

**DOI:** 10.3389/fnut.2023.1306052

**Published:** 2023-10-12

**Authors:** Renan Muniz-Santos, Peter Watt, Igor Jurisica, L. C. Cameron

**Affiliations:** ^1^Laboratory of Protein Biochemistry, The Federal University of Rio de Janeiro, Rio de Janeiro, Brazil; ^2^Environmental Extremes Lab, Sport and Exercise Science and Medicine Research and Enterprise Group, University of Brighton, Brighton, United Kingdom; ^3^Osteoarthritis Research Program, Division of Orthopedic Surgery, Schroeder Arthritis Institute and Data Science Discovery Centre for Chronic Diseases, Krembil Research Institute, University Health Network, Toronto, ON, Canada; ^4^Departments of Medical Biophysics and Computer Science, and Faculty of Dentistry, University of Toronto, Toronto, ON, Canada

**Keywords:** sportomics, doping, hypoxia, metabolomics, omics, translational medicine, computational biology, big data

Exercise is known to have a substantial impact on human metabolism, affecting metabolic fluxes and their regulation. The induced metabolic response during and following exercise can help guide our understanding of athletic performance and establish a nexus with translational results for diseases. However, the intricacies of this response can pose challenges when it comes to understanding the repercussions of sports nutrition and doping. WADA oversees the rules and implementation of anti-doping processes foe major sporting events and a small number of countries have enacted specific legislation that criminalizes sports doping. The use of anabolic steroids is most frequent in the USA, Brazil, Australia, and the Nordic countries of Europe. The greatest number of doping cases were uncovered at the Winter Olympics in Sochi, with 55 cases that resulted in 21 medals being stripped. The Russian Federation has the most anti-doping rule violations in athletics (*n* = 41), followed by Kenya and India (both with 12) and the USA (*n* = 11) (1). It should be noted that besides impacting performance, the athlete's health is also at risk (2).

Since the late twentieth century, there has been an increasing integration of “-omics” sciences in sports, starting with genomics. In 2011, we proposed “sportomics” as an “-omics” approach in sports, enabling sample collection during training and competition. We aimed to investigate metabolic responses during exercise, particularly at the highest level with professional athletes, that push the body to the physical limit and enable us to study conditions occurring during many diseases. This understanding also benefits athletic performance and recovery and provides insights into exercise-induced changes in metabolism and drug kinetics. Sportomics, therefore, may also contribute to doping control, implementation and regulation (3).

In the microbiome era, food sources are crucial to understanding the symbiotic metabolic interactions in humans. Loureiro et al. conducted a study using nuclear magnetic resonance spectroscopy and traditional biochemical analysis of serum samples. Their randomized, double-blind, crossover trial compared the effects of animal-based and plant-based protein supplements, specifically pea protein, on many metabolic parameters in a group of professional soccer players. The authors concluded that pea protein supplementation is a viable alternative to whey protein for providing essential nutrients. It is worth emphasizing that relatively few studies have compared pea protein to whey protein, mainly evaluating resistance training. This topic holds significant relevance given the growing cultural aims to decrease meat consumption and reduce carbon footprint. Importantly, the cost of pea protein is <50% that of whey protein and so is cost effective.

Exercise can be used as a model for understanding metabolic stress, for example, the effects of hypoxia-induced excitotoxicity through glutamate release in the synaptic cleft (4). This phenomenon is ultimately the underlying cause of neuronal cell death following a stroke. As highlighted in sportomics studies, it is important to collect samples during the real conditions that athletes face (in competition vs. training, environmental impact such as temperature, humidity, wind, and barometric pressure). In this sense, Post et al. conducted a study in atmospheric simulator chambers replicating normobaric hypoxia (13% O_2_), equivalent to conditions at an altitude of 3,800 m. They hypothesized that fructose supplementation could benefit individuals exposed to hypoxia. The inspiration came from the physiological adaptation observed in naked mole-rats, incredible creatures that can resist over 18 mins of anoxia and are highly resilient to various conditions. This resistance has been attributed to the rats' fructose metabolism's ability to bypass phosphofructokinase inhibition during hypoxia. Despite the logical reasoning behind their translational theory, their research found no effects of acute fructose supplementation on physical or cognitive performance in humans. This lack of improvement was observed in non-acclimatized healthy humans exposed to moderate levels of hypoxia. High-altitude sports require athletes to acclimatize before performing their activities to achieve their best performance. The hypoxia experienced at high altitudes can also induce serious health conditions, such as acute mountain sickness, lung edema, and cerebral edema. In this context, various methods have been studied to aid in acclimatization, including training at high altitudes, hypoxic generators and using pharmacological aids like acetazolamide. In this context, the Post et al. study offers insights to athletes and exercise practitioners who encounter hypoxia, such as mountaineers and individuals participating in activities in high-altitude cities worldwide. As indicated by the authors, further exploration of fructose metabolism may hold implications for addressing conditions related to oxygen deprivation, including heart failure, stroke, cancer, circulatory disorders, and neuronal excitotoxicity.

Whilst, traditionally, exercise science has focused on enhancing physical performance and preventing diseases, it has been increasingly recognized as a valuable tool for exploring how changes or stresses to exercise metabolism can provide a useful insight to examine various conditions that may affect health. For example, the concept of inducing a hypermetabolic state through exercise can provide unique insights into our understanding of human diseases, especially where metabolic processes are altered, for example such as in sepsis or other infections. As such, sportomics can leverage cutting-edge techniques such as non-targeted mass spectrometry analysis to analyze and identify metabolomic changes. Casado-Lima et al. employed an untargeted sportomics-based approach, investigating alterations in metabolism by analyzing urine samples collected during a professional soccer match. Remarkably, this research highlights unexpected parallels between the metabolic alterations induced by exercise and those seen in the inherited disorder Hawkinsinuria. Thus, beyond understanding its impact on sports performance, the study suggests exercise-induced alterations in tyrosine metabolism in non-affected people could be a potential model to investigate Hawkinsinuria.

One noteworthy insight we have gained from translating exercise findings to diseases related to hyperammonemia. Elevated ammonia concentrations (comprising ammonia and ammonium) in the bloodstream can occur during and after intense or prolonged exercise or during hepatic failures. Exercise-induced hyperammonemia is a phenomenon known to impair physical performance by causing central and peripheral fatigue. Many previous studies have been conducted to find interventions to mitigate the ammonia increase during exercise (5–12). In this context, Yang et al. conducted an *in vitro* study to explore the effects of α-ketoglutarate supplementation on various parameters associated with cell growth and metabolism, including ammonia production. Previous research has identified the potential performance benefits of α-ketoglutarate supplementation. Yang's study indicates that α-ketoglutarate supplementation effectively reduces ammonia production by over 20%. Additionally, it decreases glucose consumption and has an impact on cell growth.

Another important aspect that this Research Topic has raised concerns doping control. While we observe an increasing detection sensitivity in doping control analysis, there are still many where unintentional doping may have occurred. Recent studies have explored unexpected sources of adverse analytical findings, such as fruits and shampoos (13–15). Additionally, dietary supplements are wellknown potential sources of unintentional doping due to the risk of cross-contamination with prohibited substances or mislabeling (16). Fredrik Lauritzen and Astrid Gjelstad examined trends in dietary supplement usage by reviewing self-declarations made by athletes on over 10,000 doping control forms. These forms routinely request that athletes provide detailed information about their supplements and medication intake. The study revealed the overall frequency of supplement use and identified the most commonly used types of supplements across various sports categories. Lauritzen and Gjelstad's study contributed to our understanding of unintentional doping through contaminated supplements. Some of the elite athletes and team have been incorporating ketones into diet to provide “marginal gains” in performance (17). It is thus natural that pushing the “marginal gains” a bit further may get us over the edge to doping and doping control. Thus, the dark side of exercise science focuses on enhancing performance by not yet banned substances, or discovering masking agents that can prevent detection of banned substances. Exploring this subject in detail is beyond the scope for this special issue, and it deserves more thorough consideration.

Overall, this Research Topic has helped us to gain a deeper understanding of the multifaceted areas within the field of sports science. We have covered topics related to *in vitro* and *in vivo* interventions connected to sports performance. Additionally, we have explored how sports studies can have implications beyond the field, contributing to our comprehension of metabolic stress-related diseases and how sports and exercise can be used in translational studies. Lastly, we have provided valuable epidemiological insights into the usage of dietary supplements and doping control analysis, which are significant subjects in exercise nutrition and medicine.

## Author contributions

RM-S: Writing—original draft. PW: Writing—review and editing. IJ: Writing—review and editing. LC: Writing—original draft, Project administration, Conceptualization.
